# Atomic‐Scale Structural Modification of 2D Materials

**DOI:** 10.1002/advs.201801501

**Published:** 2019-01-22

**Authors:** Yao Xiao, Mengyue Zhou, Mengqi Zeng, Lei Fu

**Affiliations:** ^1^ The Institute for Advanced Studies (IAS) Wuhan University Wuhan 430072 P. R. China; ^2^ College of Chemistry and Molecular Sciences Wuhan University Wuhan 430072 P. R. China

**Keywords:** 2D materials, atomic defects, edge structures, grain boundaries, property tuning

## Abstract

2D materials have attracted much attention since the discovery of graphene in 2004. Due to their unique electrical, optical, and magnetic properties, they have potential for various applications such as electronics and optoelectronics. Owing to thermal motion and lattice growth kinetics, different atomic‐scale structures (ASSs) can originate from natural or intentional regulation of 2D material atomic configurations. The transformations of ASSs can result in the variation of the charge density, electronic density of state and lattice symmetry so that the property tuning of 2D materials can be achieved and the functional devices can be constructed. Here, several kinds of ASSs of 2D materials are introduced, including grain boundaries, atomic defects, edge structures, and stacking arrangements. The design strategies of these structures are also summarized, especially for atomic defects and edge structures. Moreover, toward multifunctional integration of applications, the modulation of electrical, optical, and magnetic properties based on atomic‐scale structural modification are presented. Finally, challenges and outlooks are featured in the aspects of controllable structure design and accurate property tuning for 2D materials with ASSs. This work may promote research on the atomic‐scale structural modification of 2D materials toward functional applications.

## 1 Introduction

Since the obtainment of graphene,^1^ 2D materials with the distinct physical and chemical features differing from their bulk counterparts^2^, ^3^, ^4^ have become the spotlight for material science, such as hexagonal boron nitride (h‐BN), transition metal dichalcogenides (TMDs), and black phosphorus (BP).^2^, ^3^, ^5^, ^6^ Nevertheless, pristine 2D materials with fixed properties may not meet the requirements of the versatile emerging applications. Therefore, property tuning plays an important role in the functional devices. Atomic‐scale structural modification can be one of the most effective ways to achieve the controllable property modulation. Different atomic‐scale structures (ASSs) can be naturally or intentionally formed along with the alteration of atomic configurations of as‐obtained 2D materials,^2^, ^7^, ^8^, ^9^, ^10^, ^11^, ^12^, ^13^, ^14^, ^15^, ^16^, ^17^, ^18^, ^19^, ^20^, ^21^ owing to the thermal motion and lattice growth kinetics. The appearance of the special structures can tune the electrical, optical, or magnetic properties of 2D materials due to the transformation of electronic structures, and it can also provide opportunities for new discoveries on the mechanism of 2D material synthesis.

According to the different atomic configurations, ASSs can be generally classified into four types: grain boundaries (GBs), atomic defects, edge structures, and stacking arrangements.^6^, ^22^, ^23^, ^24^, ^25^, ^26^ Researchers have devoted much effort to the formation mechanism, design strategies, and property modulation of atomic‐scale structural modification of 2D materials recently.^27^, ^28^, ^29^, ^30^, ^31^, ^32^ Some ASSs can naturally appear in as‐obtained 2D materials as intrinsic defects, and others can be introduced intentionally during the synthesis or by post treatment. In addition, the possible formation mechanism of ASSs has been studied, which offers guidance for the controllable modification of the 2D materials. Furthermore, the modulation of the electrical, optical, and magnetic properties can derive from the transformation of electronic structures, which offer access to the application extension of 2D materials.

**Figure 1:**
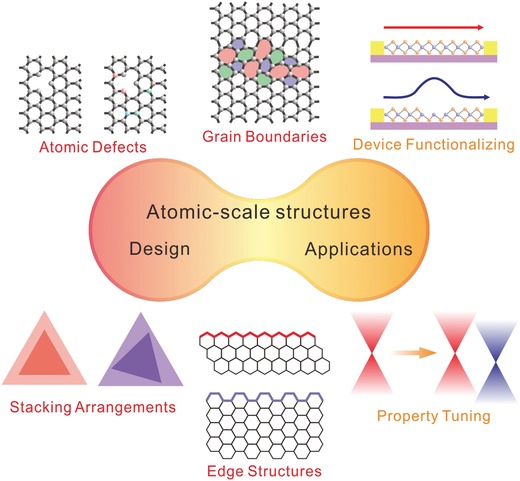
The design and application of ASSs of 2D materials.

On account of the recent progress, we systematically review the development of atomic‐scale structural modification of 2D materials. In this review, we first introduce the detailed atomic configurations of each type of ASSs and the underlying formation mechanism (**Figure** **1**). Second, we emphatically introduce design strategies for specific ASSs, especially for atomic defects and edge structures. Furthermore, the modulations of electrical, optical, and magnetic properties are presented. The modulation mechanism and some functionalized devices are also demonstrated. Finally, we conclude the problems and challenges in the design of the ASSs of 2D materials and the related application. By improving the controllability of modification, giant progress will be achieved in functionalized devices, including electronics, optoelectronics, or even integrated circuits. We expect that the deep investigations of atomic‐scale structural modification of 2D materials will facilitate the property modulation of 2D materials and enable the construction of more complex devices for practical applications.

## 2 Classification of ASSs of 2D Materials

Along with the discovery of numerous 2D materials, a diversity of ASSs has been reported. They have considerable impact on the morphology and atomic configuration of 2D materials. Herein, we classify the ASSs as GBs, atomic defects, edge structures, and stacking arrangements in accordance with atomic configurations, and expound them one after another.

GBs would appear between two domains with distinct atomic configuration from both domains, when the corresponding grains expand to adjacency with misorientation or fractional periodicity of crystal unit cell. Taking graphene as an example, incommensurate stitching of domains results in the GBs but commensurate stitching does not.^33^ The atomic configuration at the GB is highly related to misorientation angles, such as the misorientation angles of 21.8° for 5–7 periodic GBs, and 32.2° for the 5–7–5–7 ones, respectively.^34^ Unlike the atom configurations at the GBs originated from domain misorientation, symmetrical 5–8–5 periodic GBs are caused by the dislocation with the Burgers vector ***b*** = (***a***_**1**_ + *a*_**2**_)/3 along the *a*_**1**_ − *a*_**2**_ direction without any misorientation between them. Additionally, meandering and aperiodic ones do not seem to be associated with the misorientation angle or the distance of two half‐lattices.^22^, ^34^ The root lies in the different growth substrates, where periodic GBs appear in graphene grown on Cu or Ni^34^, ^35^ and aperiodic ones in graphene epitaxially grown on SiC.^36^ On the metal substrates, two different oriented half‐lattices on two different domains would form a ridge structure on the domain boundary to avoid the overlap of the two parts, where the graphene lattice could be reconstructed due to the different binding energy between metal and graphene.^35^ However, epitaxial graphene on SiC would just copy the structure of the SiC surface (both the hexagonal structure and the defects), and it could not be reconstructed on the noncatalytic SiC substrate. For polycrystalline h‐BN, tilt misorientation angles lead to polar 5–7 GBs (B‐ or N‐rich), and dislocation with Burgers vector (1,1) comprises the nonpolar 8–4 periodic one,^37^, ^38^, ^39^ where the periodic dislocations may be also induced by the reconstruction of h‐BN on the catalytic growing substrates (such as Cu^38^). Unlike graphene and h‐BN, catalysts are not necessary for TMD growth, so GBs in TMDs would form as the two domains stitch with each other, when two adjacent grains show misorientation or dislocation. Post‐synthesis modification could also result in TMD GBs due to the reconstruction of TMD structures induced by adscititious atoms.^40^ Actually, GBs in TMDs are far more complicated. Dislocations with different misorientation angles or Burgers vectors would comprise plenty of aperiodic or periodic GBs such as 5–7, 8–4, 4–6, or 6–8 rings,^41^, ^42^, ^43^, ^44^, ^45^ among which the special mirror twin boundaries (MTBs) appear at the boundaries of two mirrored domains other than those of tilt domains.^46^

Point defects in 2D materials, referring to atomic defects, can also have impact on atomic configurations. Atomic defects in 2D materials, including atomic vacancies and atomic doping, refer to the missing and substitution of the original atoms, respectively. As the intrinsic defects of crystals induced by thermal motion, atomic vacancies generally exist in as‐obtained 2D materials.^32^, ^47^, ^48^, ^49^, ^50^, ^51^ For instance, in graphene, atomic monovacancies, or divacancies are easily coalesced and reconstructed.^52^, ^53^ In single‐layer graphene (SLG), two single vacancies unite into a 5–8–5 double vacancy, and eventually reconstructed into a much more stable 555–777 defect.^54^ Similarly, atomic vacancies also appear in h‐BN after radiation, and the reconstruction proves possible to happen as well.^55^ Unlike the graphene and h‐BN, the atomic vacancy type is more abundant in metal chalcogenides,^29^, ^56^, ^57^ mainly including transition metal vacancy and chalcogen monovacancy or divacancy. Atomic doping could be another kind of atomic defects, which happens with the substitution of heteroatoms including nonmetal elements (such as B, N, P, As, chalcogens, and halogens), transition metals, and rare earth elements. The possibility of introducing dopants varies from different doping atoms. For example, N atoms can easily replace the C atoms in graphene but metal atoms may not. Atomic doping could be easy to achieve when the radius and valence electrons of doping atoms are similar to those of target material atoms, otherwise the atoms would be extremely difficult to be introduced.

Among these ASSs, edge structures have also attract much attention.^31^, ^58^, ^59^, ^60^, ^61^, ^62^, ^63^, ^64^, ^65^, ^66^, ^67^, ^68^, ^69^, ^70^, ^71^, ^72^, ^73^, ^74^ According to the atomic alignment, majority of edge structures are armchair (AC) and zigzag (ZZ) edges. For the different stability of various edge structures, the edge reconstruction could be general.^68^, ^75^, ^76^ Due to the relatively high reactivity of TMD edges, the reconstruction happens as a transition state during the chemical vapor deposition (CVD) process.^42^ For further understanding of the thermodynamic stability of different edges and edge‐state transformations, it is of great necessity to focus on the reconstruction behavior. Except for edge controlling for nanostructured or regularly shaped 2D crystals, complicated edge configuration could result in complex shapes, including patterns, dendrite‐like shapes, and fractals.

Stacking arrangements of few‐layered 2D materials are also recognized as an indispensable kind of ASSs. Due to the interesting behavior of interlayer rotations, various stacking arrangements have received considerable attention.^77^ In general, different 2D material stacks could be naturally achieved by transferring the exfoliated materials layer‐by‐layer or during the CVD processes.^78^, ^79^, ^80^ The stacking arrangement of CVD‐grown 2D materials shows dominant orientation. As a demonstration, the as‐grown few‐layered graphene (FLG) single crystals present almost only two interlayer rotations angles—0° (AB stacking) and 30° (AA stacking).^81^ As an isostructural layered material with graphene, CVD‐grown few‐layered h‐BN also shows dominant orientations of 0° (AA' stacking) and 60° (AB stacking),^27^ because the lowest energy and interlayer distance of AA' or AB stacking bilayer h‐BN has been predicted via theoretical calculation.^82^ Furthermore, for CVD‐grown bilayer MoS_2_, the main rotations angles are 0° (AB stacking) and 60° (AA' stacking), due to the smaller distance and the lowest ground‐state total energy at the twist angles of 0° and 60°.^27^, ^79^, ^83^ However, based on intentionally stacked 2D materials, more random stacking arrangement of 2D materials can be achieved by transferring one layer onto another or enfolding the materials.^80^, ^84^ The method is potential for diverse stacking arrangements of 2D materials with arbitrary twist angles, but it is less compatible with industrial production. Owing to the thermodynamical stability, few‐layered 2D materials with arbitrary twist angles are extremely difficult to obtain via conventional chemical process, which still need further development.

## 3 Design Strategies of ASSs Modification of 2D Materials

Controllable atomic‐scale structural modification is of great necessity for accurate property tuning of 2D materials, which can be achieved during the chemical synthesis or after the growth, especially for atomic defects and edge structures. Here, we would introduce the mainstream design strategies and the corresponding mechanism.

### 3.1 Design of Atomic Defects

Atomic defects can be intentionally designed and introduced to 2D materials. To achieve the controllable tuning of properties, there are many strategies for the design of atomic defects with high controllability including direct introduction and post‐treating.

#### 3.1.1 Direct Introduction

**Figure 2:**
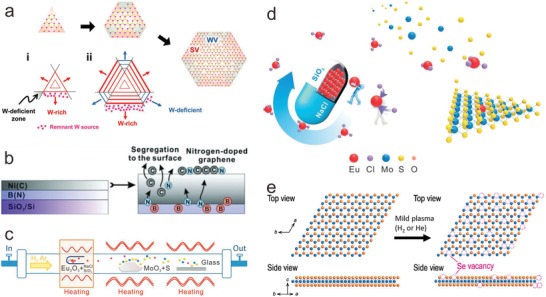
Introduction of atomic defects in different 2D materials by direct introduction and post‐treating. a) Schematic of the growth mechanism of heterogeneous defects in hexagonal WS_2_, in which blue and red regions represent facet edges for WV and SV domains, respectively. Reproduced with permission.^86^ Copyright 2017, John Wiley and Sons. b) Schematic illustrations of concurrent segregation for NG fabrication. Reproduced with permission.^87^ Copyright 2011, John Wiley and Sons. c,d) Schematic illustrations of Eu‐embedded MoS_2_ crystal synthesis via MASR CVD process. Reproduced with permission.^25^ Copyright 2018, John Wiley and Sons. e) Scheme of introduction of Se‐vacancies to WSe_2_ via H_2_ or He plasma. Reproduced with permission.^32^ Copyright 2016, American Chemical Society.

Direct introduction refers to the direct modulation of the chemical potential of each precursor during the synthesis of 2D materials, so that the reaction dynamics can be modulated to induce atomic vacancies. Sanyal et al. found that formation energy of S vacancies was relatively lower in MoS_2_, especially in the Mo‐rich conditions, so S vacancies were the richest defects in practical growth conditions.^85^ The conclusion has been also testified in other TMDs. Lee and co‐workers also did achieve hexagonal WS_2_ with heterogeneous defects (both S vacancies (SVs) and W vacancies (WVs)) by changing the S/W supply, according to the synthetic route of **Figure** **2**a.^86^ This method could be adopted for other TMDs toward different device requirements.

Atomic doping can also be achieved by introducing dopants to precursors. Because the dopants are easily involved in the synthesis process, doped materials could be obtained directly via CVD and chemical vapor transport (CVT) process. Liu and co‐workers developed a facile technique for growing N‐doped graphene (NG) using embedded C and N sources (Figure 2b). Utilizing segregation phenomenon, the B‐trapped N species as well as the trace amount of C species involved in Ni film are simultaneously squeezed out to form uniform NG film by thermal annealing.^87^ For CVT strategies, dopants are usually mixed with precursors and transport agents, which is suitable for the fabrication of most doped 2D materials.^88^, ^89^, ^90^ Similarly, atomic doping is also realized in CVD‐grown 2D materials by simply changing the component of precursors.^91^, ^92^ However, it is not effective in dealing with those which are extremely difficult to be doped in materials. Fu and co‐workers achieved rare earth element‐doped MoS_2_ for the first time via a matrix‐assisted sustained‐release CVD (MASR CVD) process (Figure 2c).^25^ SiO_2_ and NaCl not only contribute to the transformation from Eu_2_O_3_ to volatile EuCl_3_, but also produce silicate serving as the sustained release for Eu source (Figure 2d). Through such a special route, Eu^3+^ was successfully embedded in MoS_2_ in spite of the dramatic difference in covalent radius of Eu and Mo. The MASR CVD could be an efficient approach to the synthesis of rare earth element‐doped 2D materials, especially for the difficultly doped ones.

Direct introduction is an effective means of modulating the distribution of atomic defects during the fabrication of 2D materials, but it is still challenging to achieve atomically precise introduction of atomic defects.

#### 3.1.2 Post‐Treating

Post‐treating is a generally utilized method to introduce atomic defects to as‐obtained 2D materials. High‐energy plasma, ion/electron beam irradiation, or chemical media can help to introduce atomic defects to the expected area of 2D materials.^56^, ^93^, ^94^ Javey and co‐workers reported that mild He or H_2_ plasma treatment was applied to Se‐vacancy engineering in the WSe_2_ lattice.^32^ Schematic structures of Se‐deficient WSe_2_ after the plasma treatment are displayed in Figure 2e. Such an approach to introducing atomic vacancies could be appropriate for other 2D materials. Similarly, ion beam technique provides an effectual way to introduce atomic defects through manipulating the species, energy, fluences, and incident angles of the ion beams.^95^ Liu and co‐workers studied the controllable defect generation by combining effects of incident angle and energy, and they found that the ion sequentially yields or experiences processes as the ion beam energy increases, including reflection, absorption, substitution, single vacancy, double vacancies, multiple vacancies, in‐plane disorder, and transmission.^96^ Actually, directly controlling the reaction conditions could directly control the vacancy type, and the irradiation could be an efficient way to achieve controllable atomic vacancies distribution.

Moreover, atomic doping directly achieved by post‐treating is generally applicable to the exfoliated or as‐obtained materials, including plasma,^97^, ^98^ chemical treatment,^99^ and ion/electron‐beam‐mediated doping.^100^, ^101^ The doping atoms can be introduced to 2D materials through the reaction with free radicals and chemical media. By intentionally introducing C source with the help of in situ electron beam irradiation, Golberg and co‐workers obtained C doped BN nanosheets, nanoribbons (NRs), and nanotubes. The possible doping mechanism can be described that the B and N vacancies are initially produced by knock‐out ejection and then the vacancies are filled by C atoms, due to the lower formation energies of substitutional C defects at both B and N sites than the B and N vacancies.^102^, ^103^ The postsynthesis doping could also be achieved in other 2D materials, through controlling atomic species in the transmission electron microscope (TEM) chamber and choosing the appropriate electron energy.^100^ Moreover, by selecting appropriate ion beams and reaction conditions, an effective doping of 2D materials with target atoms can also be realized, such as B‐ and N‐doped graphene.^101^ The disadvantage lies in the poor controllability which may lead to the damages in 2D materials due to the high‐energy and reactive particles. Nondestructive and controllable strategies for atomic doping remain exploration and development.

### 3.2 Design of Edge Structures

As aforementioned, the edge configuration highly affects both the morphology of nanostructured 2D materials and the edge electronic structures. Hence, it is important to achieve the precise edge controlling for morphological engineering and edge‐state modulation. Actually, edge structures are closely related to growth dynamics of crystals based on the different stability of edges. Here, several strategies for the design of edge structures would be illustrated, including anisotropic etching, molecular assembly, lattice plane control, and chemical potential control.

#### 3.2.1 Anisotropic Etching

Anisotropic etching is a top‐down method to achieve edge controlling with the assistance of metal particles and reducing atmosphere. For instance, by virtue of thermally activated Ni nanoparticles, the anisotropic etching method could result in different graphene domains with different edges, including equilateral triangles, NRs, and other nanostructures with specific edges.^104^ The anisotropic etching may be promising for realizing the edge‐controllable preparation of 2D materials, on account of a significant difference in chemical activity between the AC and ZZ edges.^105^ In addition to catalytic nanoparticles cutting, hydrogen can also be used to etch materials. The fractals of graphene flakes (GFs) via the complicated etching process have been realized by using H_2_ to etch the graphene grown or exfoliated on a liquid copper surface.^106^ The repeating construction of a basic identical motif leads to the etched fractal patterns, and the physical origin of the pattern formation is consistent with a diffusion‐controlled process. Fractal degree of isolated GFs highly depends on the etching time. It proves that the diffusion‐controlled etching process may be applicable to other 2D even 3D materials.

#### 3.2.2 Molecular Assembly

Molecular assembly is commonly used for the bottom‐up synthesis of nanostructured 2D materials, especially for graphene NR (GNR).^107^, ^108^ Bottom‐up techniques are based primarily on organic synthesis beginning with small molecular modules, and then experiencing a chemical reaction to form covalently linked 2D network structure. Fasel and co‐workers reported that GNRs with various edges can be fabricated on a suitable substrate by designing appropriate precursor monomers,^23^ such as AC, ZZ or even complicated mixed edges and modified edge with different functional groups. The specific steps are as follows: 1) special monomers can be obtained by multi‐step organic synthesis and then deposited on the Au(111) single‐crystal surface; 2) monomers are activated for polymerization and then cyclodehydrogenation. Apparently, such a method is less efficient and universal for a number of 2D materials beyond graphene.

#### 3.2.3 Lattice Plane Control

**Figure 3:**
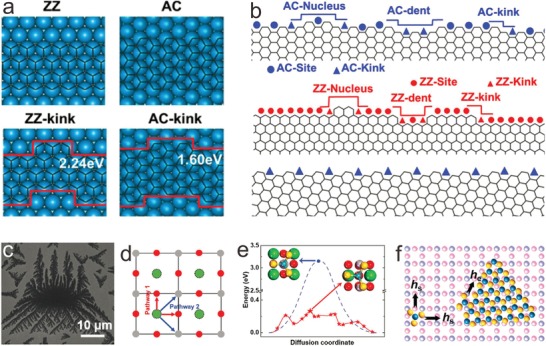
Edge controlling mechanism of lattice plane control. a) Binding energy for ZZ‐ and AC‐kink on Pt(111). b) Illustrations of kinks and nucleus on AC (upper), ZZ (middle), and 19.1° slanted (lower) edges. Reproduced with permission.^109^ Copyright 2013, National Academy of Sciences. c) Scanning electron microscopy (SEM) image of the dendritic monolayer MoS_2_ on STO(001). d,e) Schematic illustration and energy transformation of different MoS_3_ monomer hopping paths (Red: pathway 1 indicating Sr–O–Sr route; Blue: pathway 2 indicating Sr–Ti–Sr route). f) Illustration of the hopping rate of MoS_3_ between adjacent sites of STO(001) (*h*_s_) and along the MoS_2_ edges (*h*_e_). Reproduced with permission.^112^ Copyright 2016, John Wiley and Sons.

In consideration of the disadvantages of anisotropic etching and molecular assembly, controlling the lattice plane of growth substrate presents higher controllability of edge structures based on the crystal growth kinetics. For instance, graphene shows different terminating edges on different crystal plane of metal substrates. Cheng and co‐workers studied edge‐controlled growth and kinetics of graphene domains by CVD, and found that the growth of graphene depends on the edge structure.^109^ According to kinetic Wulff construction (KWC) theory, the edge with the fastest growth rate gradually disappears, and only the edges with the slowest growth rate survive during the growth process. Generally, 19.1° slanted edge grows the fastest, and the ZZ edge grows the slowest due to the highest binding energy (**Figure** **3**a,b). The different growth rate of the different edges could be attributed to the energy of the addition of carbon atoms onto the edges with kinks.^110^ However, on an isotropic substrate surface, such as liquid Cu, round graphene crystals could be obtained with mixed “sawteeth” edge with alternating ZZ and AC edges, which possibly result from similar free energy of ZZ and AC edges.^111^ In the case, the edge controlling of h‐BN on metal substrates is analogous though there are two types of ZZ edges—B‐ZZ and N‐ZZ terminations.

Additionally, fractals could also be obtained on special substrates. Zhang et al. obtained the MoS_2_ dendrites on SrTiO_3_ (STO)(001) substrate with fourfold symmetry via a facile CVD method (Figure 3c).^112^ After the as‐produced MoS_3_ monomer stably adsorbing on Sr site of STO(001), there are two different hopping paths: Sr–O–Sr and Sr–Ti–Sr (Figure 3d), where the former one needs a lower barrier (Figure 3e). It means that the MoS_3_ monomer tends to hop between Sr and O sites. It leads to different hopping rates between surface adjacent sites (*h*_s_) or along the MoS_2_ edges (*h*_e_) (Figure 3f), and then the formation of MoS_2_ fractals. It could be concluded that substrate design is an effective way to control edge structures of 2D materials.

#### 3.2.4 Chemical Potential Control

**Figure 4:**
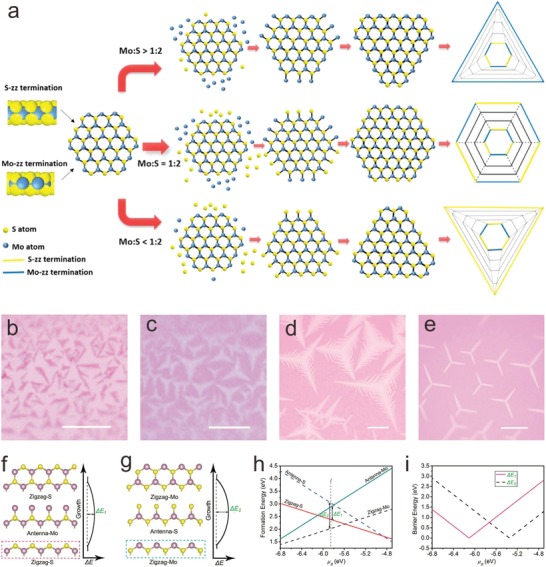
Edge structure design via chemical potential control. a) Schematic illustration of the relationship between the Mo:S atom ratio and the MoS_2_ domain shape. Reproduced with permission.^113^ Copyright 2014, American Chemical Society. b–e) Optical images of dendritic and star‐like MoS_2_ crystals grown with different S supply. Scale bars are a) 10, b) 10, c) 100, and d) 100 µm, respectively. f–g) Schematic illustrations a one‐step growth of ZZ‐S–antenna‐Mo–ZZ‐S and ZZ‐Mo–antenna‐S–ZZ‐Mo with energy barriers of Δ*E*_1_ and Δ*E*_2_, respectively. h) Edge formation energy curves and i) energy barrier (Δ*E*_1_ and Δ*E*_2_) transformation toward different sulfur chemical potentials (*µ*_S_) for the different edge configurations Reproduced with permission.^117^ Copyright 2017, John Wiley and Sons.

The chemical potential of precursors can exert great influences over edge terminations, and thereby affect the crystal morphology. For example, TMD growth follows the principle that the edge configuration depends on the growth rate in different lattice directions which is dominated by the chemical potential of precursors.^6^ Taking MoS_2_ as a demonstration, the Mo‐ZZ and S‐ZZ edges, with the lowest energy and the slowest growth rate, are the dominant edges during the growth process. Because of the distinct stability of Mo‐ZZ and S‐ZZ edges under different precursor concentration atmosphere, the relative growth rates of Mo‐ZZ and S‐ZZ edges vary from the Mo:S ratio, resulting in the different edge termination of domains. Therefore, MoS_2_ shapes change from equilateral triangular to truncated triangular, and then to hexagonal by controlling the Mo:S ratio (**Figure** **4**a).^113^, ^114^ For other 2D materials, such as GaSe, Xiao and co‐workers reported the growth‐etching‐regrowth method to fabricate GaSe with Ga‐ZZ and Se‐ZZ terminations, forming the AC edges and tilted ZZ edges by controlling chemical potentials.^115^

It is aforementioned that different edge structures for simple Euclidean shape are attributed to different edge terminations. Complicated shapes with mixed edge configurations, especially for fractals, can mainly result from complex expanding behavior on 2D crystal edges. Despite the fact that there are a lot of challenges in tailoring the shapes of patterned 2D materials due to the extensive complicated atomic‐level processes, it is possible to achieve the shape controlling from negative to zero to positive curvature via controllable nonequilibrium growth.^116^ Initially, dendritic structures could be observed at higher precursor concentrations.^114^ Liu and co‐workers demonstrated GFs shape evolution with the edge curvature changing by controlling the ratio of Ar:H_2_ during the CVD process on liquid Cu surface.^116^ As Ar:H_2_ ratio is increased, the edges of the regular hexagon gradually curve to the center. Similarly, by controlling the precursors, MoS_2_ fractals were also successfully obtained, while the mechanism could be complicated. Loh and co‐workers achieved the shape transformation of MoS_2_ from compact triangles to fractal flakes or even star‐like crystals via reduced S supply (Figure 4b–e).^117^ The mechanism is described as a competing growth for the different barriers of the ZZ‐S–antenna Mo–ZZ‐S and ZZ‐Mo–antenna S–ZZ‐Mo routes (Figure 4f–i). Terminal‐atom‐dependent edge growth rates and alternatively growth of ZZ‐S or ZZ‐Mo edges in three directions finally result in three‐level MoS_2_ fractals. Moreover, diverse MoS_2_ fractals were realized coinstantaneously by deliberate introduction of twin defects and different local ratio of Mo:S vapor down the stream.^118^ To sum up, chemical potential control can be one of the most significant strategies to achieve the design of edge structures with high controllability. It is a promising method to be expanded to the design of edge structures for other materials.

## 4 ASS‐Engineered Band Structure

**Figure 5:**
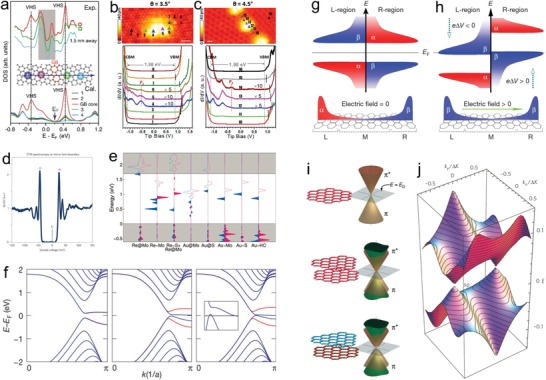
Band structure tuning engineered by ASSs of 2D materials. a) VHS shown both in experimental (the upper panel) and calculated (the lower panel) DOS results of the GB of graphene. The middle panel displays the model for theoretical calculation. Reproduced with permission.^122^ Copyright 2014, American Physical Society. b,c) STM images (upper panels) with marks and the corresponding d*I*/d*V*–bias STS curves (lower panels) of two different domains near defect cores of low‐angle GBs of WSe_2_. Reproduced with permission.^121^ Copyright 2016, American Chemical Society. d) The d*I*/d*V* STS of a MTB on the monolayer MoSe_2_, showing a bandgap of 73 meV, where 0 V sample bias, *Ψ*_−_, and *Ψ*_+_ represent *E*_F_, edge state below and above the gap, respectively. Reproduced with permission.^119^ Copyright 2016, Springer Nature. e) LDOS of the Re and Au dopant atoms at eight considered atomic structures. Reproduced with permission.^88^ Copyright 2014, John Wiley and Sons. f) Spin‐resolved band structures of a 16‐ZGNR with *E*_ext_ = 0.0 (left), 0.05 (middle), and 0.1 V Å^−1^ (right), respectively. The red and blue lines denote bands of α‐spin and β‐spin states, respectively. Inset in panel right shows the band structure in the range |*E*–*E*_F_| < 50 meV and 0.7π <*k* < π (the horizontal line is *E*_F_). g) Schematic diagram of DOS of a ZGNR without a *E*_ext_. Panel top shows the same bandgap of the occupied and unoccupied localized edge states (α‐spin and β‐spin) on the left side (L‐region as defined at the bottom) and the right side (R‐region). Panel bottom shows the spatial spin distribution of the highest occupied valence band states. h) Schematic diagram of DOS of a ZGNR with a *E*_ext_. Panel top shows the lowered electrostatic potential on the left edge (eΔ*V* < 0) and the raised one on the right edge (eΔ*V* > 0) with a transverse *E*_ext_. Panel bottom shows the resulting states being only β‐spin at *E*_F_. Reproduced with permission.^24^ Copyright 2006, Springer Nature. i) Electronic structures of a SLG, a Bernal stacking BLG, an asymmetric BLG. Reproduced with permission.^134^ Copyright 2006, The American Association for the Advancement of Science. j) Dispersion of lowest energy states in a BLG with a twist angle of 1.79°, in which two saddle points (masked as sp) appear in both negative and positive energy bands, known as VHSs. Reproduced with permission.^132^ Copyright 2010, Springer Nature.

Atomic‐scale structural modification can directly affect the localized electron distribution. Owing to the different atomic configurations, band structures can be efficiently engineered and density of states (DOS) can be tuned. Initially, the existence of GBs could lead to dramatic differences in the electronic band structures of 2D materials.^119^, ^120^, ^121^, ^122^ Hou and co‐workers observed van Hove singularities (VHSs) at the GBs of graphene.^122^ As a demonstration, **Figure** **5**a shows the experimental scanning tunneling spectra (STS) and the simulation of the 5–7 periodic GBs, in which VHSs are only observed at the GBs. In addition to WSe_2_ with low‐angle GBs, differences in d*I*/d*V* STS results also appear at the points lying on GBs, referring to point 4–6 in Figure 5b and point 12–14 in Figure 5c, respectively.^121^ Namely, multiple deep gap states are observed near the dislocations cores, located above the Fermi level and close to the conductive band minimum (CBM) without occupation. The valence band maximum (VBM) is also noted to shift away from the Fermi level, and the maximum of the shift is reached at the position near the dislocation cores. Moreover, as shown in Figure 5d, an energy bandgap of 73 meV with sharp peaks at the band edges could be observed in the d*I*/d*V* STS result of monolayer MoSe_2_ at 4.5 K.^119^ For topological insulators (TIs), such as Bi_2_Te_3_, twin boundaries could alter the electronic structures, which result in the new occupied states and the increase of DOS near CBM.^123^ In brief, the dislocations and distortions at the GBs result in the electron delocalization of the conjugated system so that new occupied or unoccupied states appear and contribute to phase transition at low temperature or special charge transport. It would be inspiring for the structure tuning and constructing on the basis of GBs of pure 2D materials.

It was reported that the introduction of atomic defects into 2D materials can also change DOS. In TMDs systems, the defect state appears at the valence band edge and inside the bandgap toward the conduction band side, resulting in Fermi level shifts.^124^ Furthermore, atomic doping can also result in band structure tuning.^92^, ^97^, ^125^, ^126^, ^127^ Suenaga and co‐workers demonstrated different local DOS (LDOS) around the dopant atoms in Re‐ and Au‐doped MoS_2_.^88^ Figure 5e shows that Re dopants add additional electrons to the conduction band of MoS_2_, and Au dopants also lead to similar mid‐gap states. In conclusion, atomic defects can modulate the band structures by introducing impurity energy levels.

Edge structures can have impact on band structures of 2D materials as well. Graphene with different edges shows distinct electronic structures, which are theoretically predicted to be related to the GNR width.^65^, ^128^, ^129^ Plenty of theoretical and experimental studies have demonstrated opened electronic bandgaps in GNRs. The bandgap originates from the edge decisive effect and quantum confinement in AC‐GNRs, while it is attributed to the hexagonal lattice's interlaced sublattice potential induced by edge magnetization in ZZ‐GNRs.^130^, ^131^ Furthermore, it was reported that ZZ‐GNRs shows semimetallic under the in‐plane homogeneous external electric fields (*E*_ext_) crossing the ZZ‐GNRs,^24^ because the conduction and valence edge‐state bands associated with α‐ or β‐spin orientation would close or enlarge the bandgap, respectively (Figure 5f). Moreover, the opposite spin states are located at the opposite sides of the ZZ‐GNR, and the influence of *E*_ext_ on them is opposite. Furthermore, the unoccupied and occupied β‐spin states (α‐spin states) are moved closer (apart) in energy (Figure 5g–h).

Different stacking arrangements of few‐layered 2D materials provide with abundant physical phenomena concerning their band structures.^132^, ^133^ Because the graphene band structures are sensitive to the lattice symmetry, the broken symmetry and interlayer coupling of Bernal stacking bilayer graphene (BLG) could lead to two additional valley splitting π and π* states, and two lower energy bands. Hence, an energy gap appears between the two low‐energy bands in twisted stacked bilayers with nonequivalent individual graphene layers (Figure 5i).^134^ Different from AB‐stacked bilayer, twisted BLG causes a considerable electron–hole asymmetry which appears in the electronic states close to the saddle points. Large twist angles lead to the saddle points far away from the Dirac cones, maintaining the single‐layer Dirac dispersion. With the twist angle decreasing, the two Dirac cones move to each other, and the low‐energy dispersions could be amended by the proximity to the saddle points.^135^ Moreover, new electronic states are also acquired in energy bands of FLG. For instance, the relative position of VHS and Fermi energy level can be tuned by varying the rotation angle in twisted graphene layers. Two symmetric rotation‐induced VHSs are ascribed to the intersection and hybridization of two Dirac cones near the center of twisted BLG Brillouin zone (Figure 5j).^132^ This research brings an interesting prospect for VHSs engineering of electronic phases. In addition, Chen and co‐workers demonstrated lower thermal conductivity in twisted BLG than that of AB‐stacked BLG.^136^ The root cause lies in the confined transport of acoustic phonons in twisted BLG. Meanwhile, Balandin and co‐workers reported that the phonon specific heat highly depends on the twist angle of the twisted BLG, especially at low temperature.^137^ Therefore, thermal performance of layered materials could also be tuned by the phonon engineering of twisted 2D atomic crystals. For few‐layered TMDs, such as MoS_2_, direct gap of the monolayer one could be transformed into indirect gaps which could be enlarged by more than 100 meV through reducing the interlayer coupling.^84^

Atomic‐scale structural modification of 2D materials can result in the modulation of band structures of materials, deriving from different atomic configurations. The transformation of band structures can serve as excellent platforms for fundamental physical research.

## 5 ASS‐Induced Property Tuning

ASSs of 2D materials exhibit different band structures, which can lead to novel properties and applications. The modulated bandgaps, mid‐gap energy levels, and spin‐dependent DOS can exert great influence on modulating electrical, optical, and magnetic properties of 2D materials.

### 5.1 Electrical Property Tuning

**Figure 6:**
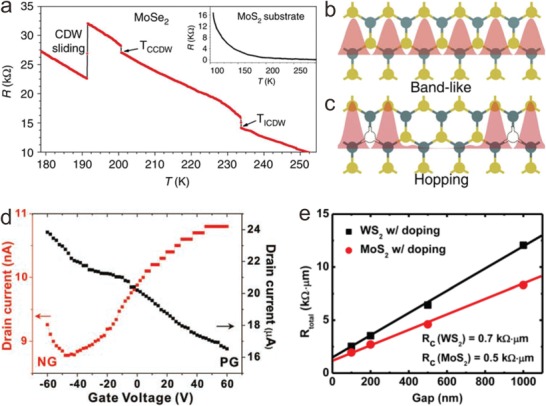
Modulation of electrical properties induced by ASSs of 2D materials. a) Temperature‐dependent resistance of MoSe_2_, showing the transitions of incommensurate CDW (ICDW) and commensurate CDW (CCDW) at 235 and 205 K, and the CDW sliding below the transition temperature. The inset shows the temperature‐dependent resistance of the bare MoS_2_ substrate without any transitions. Reproduced with permission.^139^ Copyright 2017, Springer Nature. Schematics of electron transport mechanism in b) perfect and c) defective MoS_2_ showing band‐like and hopping behavior, respectively. Reproduced with permission.^29^ Copyright 2013, Springer Nature. d) Drain–source current–gate voltage (*I*_ds_–*V*_g_) curves of NG (N/C 1.6 at%) (red) and pristine graphene (PG) (black). Reproduced with permission.^87^ Copyright 2011, John Wiley and Sons. e) Reproduced with permission.^98^ Copyright 2014, American Chemical Society.

It is aforementioned that band structure tuning can lead to the transformation of charge transport and the appearance of impurity energy levels of 2D materials. These can have impacts on the electrical properties. As line defects, GBs may lead to low carrier mobility or conductance of 2D materials.^22^, ^46^, ^138^ Even so, unusual electrical properties can also be induced by GBs, such as the giant resistance transformation attributed to charge density wave (CDW) phase transition. As a demonstration, monolayer MoSe_2_ with MTBs shows two resistance jumps at ≈235 and ≈205 K, which are attributed to the incommensurate and commensurate CDW transitions,^139^ as shown in **Figure** **6**a. The root cause lies in the Peierls transition at a low temperature. The large drop of resistance at lower temperature indicates the depinning of CDW from the defects, corresponding to the CDW sliding in Figure 6a. For the temperature‐dependent resistance of the MoS_2_ substrate without any resistance jumps (Figure 6a inset), the resistance jumps and the large drop are attributed to the phase transition of MoSe_2_ with MTBs. This may provide a promising application for low‐energy electrical transport.

Atomic defects can also affect the charge transport of 2D materials owing to the different charge density of atomic vacancies or doping atoms. For example, the charge transport of few‐layer MoS_2_ was proved to be hopping through SV‐induced localized states (Figure 6b,c),^29^ indicating that the carriers could still transport at the certain defects. Similarly, Fu and co‐workers demonstrated the 2D variable range hopping (VRH) behavior of as‐grown 1T′‐MoTe_2_, which may also indicate the atomic vacancies lying in such a metastable TMD with the distorted structure.^19^ Unlike atomic vacancies, atomic doping can directly modulate the carrier transport by introducing different energy levels. Due to the Fermi level shifting of external electron carrier doping, atomic doping would introduce donor or acceptor levels in the original band structures, which cause the distinct carrier transfer characteristics.^140^, ^141^ For instance, NG enables the transformation from pristine zero‐bandgap graphene to a n‐type semiconductor, as shown in Figure 6d.^87^ Furthermore, electrical properties vary from the doping atoms. Taking BP as an example, isovalent doping retains anisotropic transport properties and semiconducting characteristics, while group IV A and VI A atoms doping would generate metallic properties.^142^ Additionally, Zhang and co‐workers studied electrical properties of the BP systems doped within the fourth‐period main group elements through first‐principles calculations.^143^ The results show that the direct bandgap of pristine BP transforms into the indirect ones, and an extra hole‐ or electron‐doping can induce a p‐ or n‐type BP respectively. In detail, the Ca‐, Ge‐, and Se‐doped BP systems are metallic, whereas the K‐, Ga‐, As‐, and Br‐doped ones are semiconductors. Therefore, the conclusion can be drawn that dopants with even valence electrons can convert BP from semiconductor to metal but those with odd valence electrons cannot. Atomic doping is also advantageous to improve the contacts between electrodes and channel materials. Ye and co‐workers demonstrated that the contact resistance (*R*_c_) of Ni‐MoS_2_ and Ni‐WS_2_ contacts decreased to 0.5 and 0.7 kΩ µm by Cl doping (Figure 6e).^98^ Here, Cl doping contributes to a large electron‐doping density and a prominent reduction of Schottky barrier width. Atomic doping plays an important role in modulating charge transport, carrier density and surface state, and it promotes promising application for functional and high‐performance devices.

### 5.2 Optical Property Tuning

Owing to bandgap engineering induced by atomic‐scale structural modification, the excitation of electrons, recombination of excitons, and the light emission processes can be modulated. Hence, optical properties of 2D materials can also be tuned. The GBs could result in special optical properties, such as the intense photoluminescence (PL) emissions. For instance, along the GBs of WS_2_ domains with localized electronic states, main PL emission is caused by trions or biexcitons rather than neutral excitons so the PL intensity is enhanced.^144^

**Figure 7:**
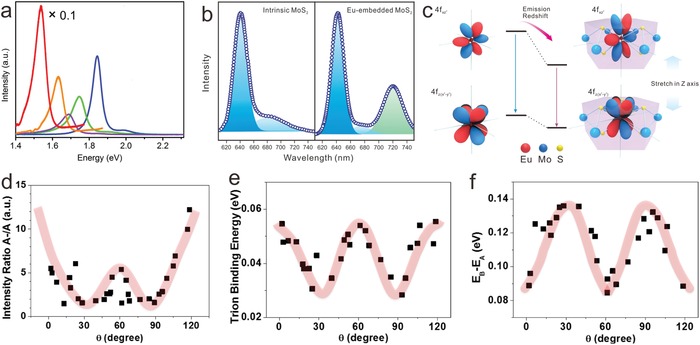
Modulation of optical properties induced by ASSs of 2D materials. a) PL spectra of pristine MoS_2_ (blue), MoS_1.4_Se_0.6_ (green), MoS_1_Se_1_ (purple), MoS_0.5_Se_1.5_ (orange), and MoSe_2_ (red), respectively. Reproduced with permission.^91^ Copyright 2014, American Chemical Society. b) Low‐temperature PL spectra of intrinsic MoS_2_ (left panel) and Eu‐embedded MoS_2_ (right panel), demonstrating a large enhancement of 723 nm peak in right panel induced by 4f–4f transition of Eu^3+^. c) Illustration of the PL mechanism and the distortion of 4f orbitals in the Eu‐embedded MoS_2_. Reproduced with permission.^25^ Copyright 2018, John Wiley and Sons. d) the intensity ratio of A^−^ trions and A excitons, and e) trion binding energy to show the interlayer coupling strength. f) Twist angle‐dependent energy difference of B and A excitons to show the interlayer coupling strength and the stability indirectly. Reproduced with permission.^80^ Copyright 2014, American Chemical Society.

As for atomic defects, the position of the defect state in the gap also influences the optical properties. It is theoretically calculated that optical transitions could occur at ≈1.0 eV for defect states, which can be beneficial for light emitting devices.^124^ PL intensity could be sensitive to the n‐doping resulting from chalcogen vacancies for TMDs in general.^145^, ^146^ Because these vacancies could result in bound excitons, the PL intensity of the excitons decreases. On account of atomic doping‐induced band structure transformations, doped 2D materials also exert distinctive optical properties. Ajayan and co‐workers achieved the tunable MoS_2_ optical bandgap between 1.85 and 1.60 eV by controlling the Se doping concentration (**Figure** **7**a).^91^ That is, atomic doping could induce a large change of the band structures, where the optical bandgaps could be tuned for a large range. In addition to tuning the properties of the subject material, doping can also alter the structure of the doped elements itself. Fu and co‐workers illuminated that 4f orbitals of Eu^3+^ embedded in MoS_2_ were stretched along the *z*‐axis in the MoS_2_ crystal‐field, resulting in the tailored PL for Eu^3+^ as shown in Figure 7b,c.^25^ Compared to intrinsic MoS_2_, a significantly enhancement could be observed in the low temperature (at 5 K) PL spectrum of Eu‐embedded MoS_2_, attributed to the 4f–4f transition of Eu^3+^. Atomic doping not only helps with the bandgap tuning of 2D materials, but also achieves the energy level splitting of dopant atoms (such as lanthanide ions), which is promising for advanced photodetectors.

Furthermore, stacking arrangements can also induce the optical property tuning of 2D materials. With the bandgap reaching the infrared range, BLG has great potential in novel nanophotonic devices for infrared light generation and detection.^147^ Dresselhaus and co‐workers studied the interlayer coupling of twisted bilayer MoS_2_ by the first‐principles density functional theory (DFT) calculations.^80^ They found the interlayer coupling reached to the maximum at 0° and 60°, and the minimum at θ = 30° and 90°, respectively. Additionally, the interlayer coupling plays a role in the PL emission variation of the twisted bilayer MoS_2_, which is attributed to the concentration of excitons and trions, and their binding energies variation with the twisted angle bilayer MoS_2_ (Figure 7d–f).

To sum up, optical property tuning is ascribed to the bandgap engineering and the charge distribution transformations induced by ASSs of 2D materials. The modulation of optical properties induced by atomic‐scale structural modification can provide much inspiration for further luminescent devices or optoelectronics.

### 5.3 Magnetic Property Tuning

**Figure 8:**
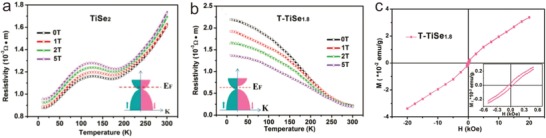
Magnetic properties induced by modified ASSs of 2D materials. The temperature‐dependent resistivity under various magnetic fields from 10 to 300 K for a) TiSe_2_ and b) T‐TiSe_1.8_. c) *M*–*H* curves at 300 K for T‐TiSe_1.8_ nanosheet, in which the inset shows the enlarged *M–H* section in the low applied field. Reproduced with permission.^150^ Copyright 2017, John Wiley and Sons.

Magnetism, which is linked to the different electronic structures for different spins, could also be tuned on account of the modulated DOS. Atomic vacancies are introduced to induce the magnetism for 2D materials, which has been theoretically predicted and experimentally achieved in graphene and MoS_2_.^148^, ^149^ Recently, Xie and co‐workers achieved the transition from metallic TiSe_2_ to high spin polarized semimetallic T‐TiSe_1.8_ for the first time, by introducing dual native defects with single Ti‐atom intercalation and Se‐anion defects.^150^ As shown in **Figure** **8**a,b, the resistivity of pristine TiSe_2_ increases toward the rising temperature, but the T‐TiSe_1.8_ exhibits an opposite behavior. It is attributed to different DOS for different spin at the Fermi level of T‐TiSe_1.8_ (the inset of Figure 8b), while metallic TiSe_2_ shows the same DOS for different spin at the Fermi level (the inset of Figure 8a). In Figure 8c, the T‐TiSe_1.8_ shows the inherent room temperature ferromagnetism according to emblematical S‐shaped field‐dependent magnetization (*M–H* curve) with significant hysteresis behavior. Else, magnetic properties also derive from atomic doping. For instance, the N‐doped graphene triggers ferromagnetism, which is attributed to delocalized electrons occupying narrow peaks at the Fermi level (*E*_F_). N‐doping generating the ferromagnetism state leaded to a strong *p*_z_ electron peak at the Fermi level in the electronic structure according to the partial DOS.^151^ Moreover, the TMDs doped Gd^3+^ with paramagnetic could use for the cancer theranostics, combining with some unique techniques.^152^, ^153^

Different edge structures can also bring about magnetic properties. TMDs with different edge configuration demonstrate different edge‐state band structures. Similar to GNRs, ZZ‐MoS_2_ NRs show metallic and ferromagnetic behaviors but AC‐MoS_2_ NRs are semiconducting and nonmagnetic, according to first‐principles calculation.^154^ Moreover, the edge magnetism is sensitive to *E*_ext_ and external pressure.

In conclusion, magnetic properties induced by atomic‐scale structural modification of 2D materials originate from the transformation of spin‐dependent DOS. These properties can open up the platforms of material magnetism for smart magnetic switches or sensors.

## 6 Summary and Outlook

Property modulations of 2D materials toward functionalized devices have attracted extensive attention in recent years. Plenty of efforts have been devoted to atomic‐scale structural modification of 2D materials and the induced property tuning as well as the relative application extension. In this review, we first introduce the main types of ASSs in terms of the different atomic configurations, including GBs, atomic defects, edge structures, and stacking arrangements. Then, to achieve the controllable property tuning, accurate atomic‐scale structural modification can be very important. The atomic defects can be introduced by direct introduction during the synthesis and the post‐treating, while the edge structures can be designed by the anisotropic etching, molecular assembly, lattice plane control, and chemical potential control. At last, we demonstrate that the modulations of electrical, optical, and magnetic properties of 2D materials are achieved by atomic‐scale structural modification. The root cause lies in the transformation of electronic structures induced by the variation of the ASS. Property tuning based on atomic‐scale structural modification can be crucial for the construction of functionalized device.

However, there are several challenges that need to be overcome. First, the universality of the existing strategies for atomic‐scale structural modification of 2D materials is urgent to be further improved, which will lay the foundations for the more extensive device application. So far, the atomic‐scale structural modification has been restricted in conventional 2D materials, such as graphene, h‐BN, traditional TMDs (MX_2_, M = W, Mo, and X = S, Se) and BP. Novel materials with ASSs will enable more exciting and interesting properties, thus offering the promising platforms for fundamental research and practical applications. Therefore, universal methods of atomic‐scale structural modification to 2D materials need to be further explored. Second, after atomic‐scale structural modification, the fidelity of the structures and properties of 2D materials needs to be optimized. The existent direct modification strategy during the fabrication is still challenging for as‐obtained 2D materials due to the possible reduction of their crystallinity or the unexpected phase transition, and the post‐treatment may lead to the degradation of some metastable materials. These can result in the giant variation of the property of 2D materials, which is contrast to the premise that atomic‐scale structural modification should effectively tune or extend the property of parent materials but not significantly destroy the intrinsic structure of the parent 2D materials. Thirdly, the precise atomic‐scale structural modification of 2D materials is also challenging. The introducing of atomic defects with precise positions can be promising to provide accurate localized property tuning, such as the LDOS, local charge density, and local charge transport. Nevertheless, the nonlocalizability and poor efficiency of the existent methods such as electron beam irradiation restricts their practical application. Fourthly, the formation mechanism of ASSs is still lack of deep understanding. In situ observation of the evolution of these structures in 2D materials via the dynamic high‐resolution characterization technique, such as TEM or scanning tunnel microscope (STM) can offer the direct evidence to show the formation process of atomic‐scale structural modification of 2D materials. Theoretical calculations can facilitate the understanding of the related formation mechanisms. The further understanding of the mechanism can become the guidance for adjusting the ASSs in 2D materials. Finally, practical applications based on 2D materials with their properties tuned by atomic‐scale structural modification still need further studies, such as the integrated electronics, optoelectronics, and magnetic devices.

2D materials with specially designed ASSs can be excellent candidates for various functional applications. The atomic‐scale structural modification can play an important role in the property tuning of 2D materials and the related device construction. It holds the prospect for structure engineering to improve the diversity of materials toward fundamental physical research, integrated circuits, high‐performance sensors, and energy transformation devices. We are convinced that the atomic‐scale structural modification will further promote the development of 2D material science.

## Conflict of Interest

The authors declare no conflict of interest.
